# The Modern Hospital in Metaphors

**Published:** 1913-06-21

**Authors:** 


### THE MODERN HOSPITAL IN METAPHORS.

Dr. Arthur E. Giles, who also responded, said :

When we speak of a hospital many different ideas are no doubt suggested to different minds. To the mean mind a hospital is a means of evading the fees of the family doctor and obtaining advice and medicine for nothing; to the narrow mind it is an institution supported by the blindly benevolent to enable callous scientists, for their own ends, to experiment on the helpless and deluded poor; to the prospective patient it means a "fearful leap in the dark," if he is the first of his circle of acquaintance to go there, or a much desired haven of refuge if his friends have been there before him. To the ubiquitous "man in the street" it is an abode of women most becomingly dressed in attractive uniforms, and called nurses; or a means of placating Providence at a cost of 5s. a year, in case his own turn should come one day; or it is what the irreverent would term a form of "fire insurance"; or again it may be just a hospital and nothing more.

Those who know hospitals from the inside, their aims, methods and management, know that a hospital is a very complex affair, composed of many parts fulfilling many functions, but all working together to one harmonious end. The whole forms one body corporate, and may be compared to a skilled artificer engaged on some valuable clock or piece of furniture or complicated piece of machinery, in repairing the ravages and damages wrought by time or accident, wear and tear. Sometimes we are engaged in regulating the mechanism, or the parts want cleaning, or the valves of the pump may be deficient, or the air-inlet to the carburettor is choked up, or the joints of the machine are working stiffly, or we are busy riveting some old piece that is badly cracked (a pursuit more particularly engaged in at mental hospitals), or haply we are restoring antiques.

If we mildly indulge in what Mrs. Browning calls "leaning o'er the window-sill of metaphor," we may recognise some of the component parts of our artificer. The brain, with its thoughts, plans, and counsels is clearly represented by the noble Chairman and the Board of Governors; the directing will of the concern is undoubtedly our urbane director and secretary, Mr. Drewett. The hands that are concerned in doing the repairing are the medical and surgical staff; and I cannot help thinking that the gentle and delicate fingers are the nursing staff. The feet that support the whole body, and without which we should go uncommonly lame, are the domestic staff, the porters, maids, scrubbers, whose work, if less showy, is none the less honourable and necessary.

Now, a body requires sustenance if it is to carry out its functions. Has the hospital a receptacle for taking in supplies? Has it what Oliver Wendell Holmes elegantly calls "a food-receiving feature, three times daily patent"? I feel sure that you here recognise an almost exact description of our treasurer; and I may add that he always leaves off with an appetite, and has not the slightest objection to snacks between meals. It were arrogant not to acknowledge that above and beyond our artificer stands the Master-Artificer, whether we call it the *Vis medicatrix naturæ* or use a higher name; compared to which or to whom our artificer himself is little more than a skilfully devised instrument, only a shade more complex than his own tools, for to quote one of the profoundest utterances of our greatest poet :

> "Nature is made better by no mean,
> But Nature makes that mean; so, over that art
> Which you say adds to Nature, is an art
> That Nature makes."

I am, however, reminded of the existence of that most worthy institution, the Society for the Prevention of Cruelty to Similes; and I will therefore cease maltreating this one, and hope that my offence has not yet rendered me liable to prosecution.

In returning thanks for the hospital, one's thoughts naturally turn to the many generous givers whose gifts make the work of the hospital possible. It may perhaps seem to some of them that giving to the hospital is like pouring water into a can with a large hole in it; they pour and pour and yet it is never full. May I say, for the comfort of all such, that in the case of the hospital the outflow is not the unaltered inflow, as it would be were it merely the wasteful overflow of extravagance, but the streams of gold and silver that are poured into it become transmuted by the alchemy of medicine and of mercy into a veritable stream of healing, perhaps markedly radio-active, and distinctly flavoured with the milk of human kindness? Although in the hospital "all are but parts of one stupendous whole," I must return thanks more particularly on behalf of the medical staff. We are sometimes apt to receive more than our share of the credit for the good results obtained; and, like the conscientious conductor of an orchestra, who refuses to accept the applause as a personal tribute, I feel that we must bring before you to share in your thanks our two invaluable helpers, the nursing staff, without whom our work in the hospital would be of no avail, and the Ladies' Association, who, by their Samaritan and Convalescent Home work complete our own.

